# Antimetastatic Therapies of the Polysulfide Diallyl Trisulfide against Triple-Negative Breast Cancer (TNBC) via Suppressing MMP2/9 by Blocking NF-κB and ERK/MAPK Signaling Pathways

**DOI:** 10.1371/journal.pone.0123781

**Published:** 2015-04-30

**Authors:** Yuping Liu, Pingting Zhu, Yingyu Wang, Zhonghong Wei, Li Tao, Zhijie Zhu, Xiaobo Sheng, Siliang Wang, Junshan Ruan, Zhaoguo Liu, Yuzhu Cao, Yunlong Shan, Lihua Sun, Aiyun Wang, Wenxing Chen, Yin Lu

**Affiliations:** 1 School of Pharmacy, Nanjing University of Chinese Medicine, Nanjing, 210023, China; 2 Medical College of Yangzhou University, Yangzhou, China; 3 Jiangsu Collaborative Innovation Center of Traditional Chinese Medicine (TCM) Prevention and Treatment of Tumor, Nanjing University of Chinese Medicine, Nanjing, 210023, China; Stanford University School of Medicine, UNITED STATES

## Abstract

**Background:**

Migration and invasion are two crucial steps of tumor metastasis. Blockage of these steps may be an effective strategy to reduce the risk. The objective of the present study was to investigate the effects of diallyl trisulfide (DATS), a natural organosulfuric compound with most sulfur atoms found in garlic, on migration and invasion in triple negative breast cancer (TNBC) cells. Molecular mechanisms underlying the anticancer effects of DATS were further investigated.

**Methods and Results:**

MDA-MB-231 cells and HS 578t breast cancer cells were treated with different concentrations of DATS. DATS obviously suppressed the migration and invasion of two cell lines and changed the morphological. Moreover, DATS inhibited the mRNA/protein/ enzymes activities of MMP2/9 via attenuating the NF-κB pathway. DATS also inhibited ERK/MAPK rather than p38 and JNK.

**Conclusion:**

DATS inhibits MMP2/9 activity and the metastasis of TNBC cells, and emerges as a potential anti-cancer agent. The inhibitory effects are associated with down-regulation of the transcriptional activities of NF-κB and ERK/MAPK signaling pathways.

## Introduction

**Fig 1:**
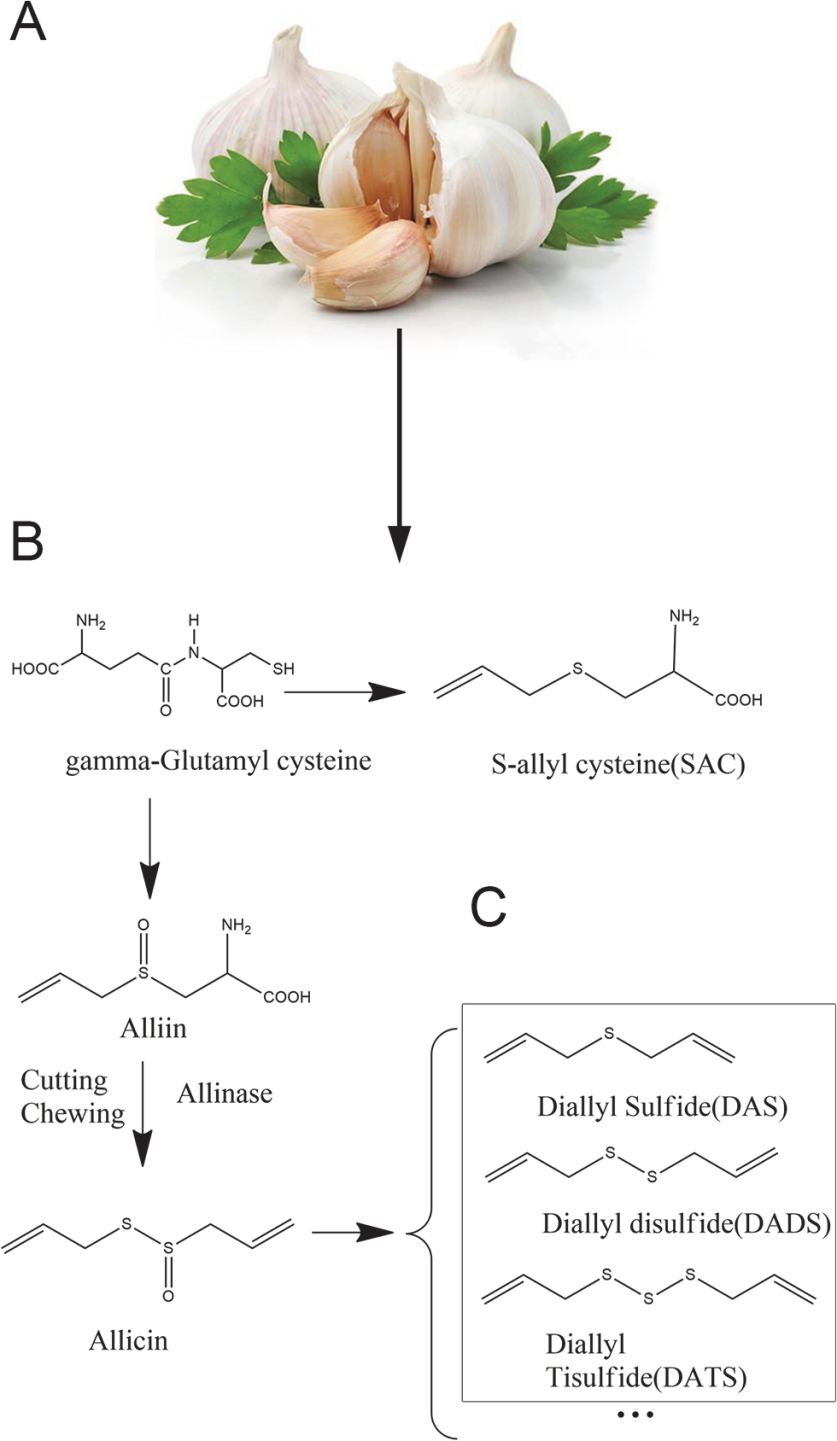
Chemical structure of garlic OSCs. (A) The plant of garlic. (B) The process of OSCs produced. (C) Chemical structure of DAS/DADS/DATS.

Garlic (*Allium sativam L*.) is renowned for the "medicine food homology" superiority in traditional and alternative medicine. Epidemiological researches have supported a positive association between dietary intake of Allium vegetables and lower cancer risk [1]. For example, meta-analysis of the association between raw and/or cooked garlic intake and the risk of colorectal cancer revealed a relative risk estimation of 0.69 [2]. Organic sulphur compounds (OSCs), the major active ingredients of garlic, have attracted great attention recently as a novel pool of cancer prevention and treatment agents. OSCs mainly include diallyl sulfide (DAS), diallyl disulfide (DADS), diallyl trisulfide (DATS), and other allyl polysulfides (Fig 1) [3]. Furthermore, studies postulate that the number of sulfur atoms accounts for the significance of biological activity of OSCs, and DATS containing the most sulfur atoms has been demonstrated to be the most effective compound[4,5].

Recent studies have demonstrated anticancer effects of DATS against breast cancer [6–8]. Breast cancer represents a top leading cause of death related to cancer in women worldwide. It has risen to 522,000 deaths by the end of 2012, with 1.7 million new cases diagnosed each year [9]. Importantly, triple-negative breast cancer (TNBC) is the most invasive and aggressive among the breast cancer subtypes. So far there is no clinical therapy specific for patients with TNBC [10,11]. Therefore, identifying biological markers of TNBC progression and finding plant resource used as both medicine and food which target the markers could be meaningful for the prevention of breast cancer metastasis, providing new therapeutic strategies for the disease.

The invasion and metastasis of breast cancer is proceeding by multiple genes and follows complex steps [12]. One of the first steps is the degradation of the extracellular matrix. During this process, matrix metalloproteinases (MMPs) play an important role and have been viewed as a promising target in tumor therapy. MMPs are over-expressed during tumor growth, invasion and metastasis and are associated with malignant tumors and an adverse prognosis in breast cancer patients [13,14]. Therefore, repression of MMPs expression and secretion may be an effective strategy for preventing cell migration and invasion. Multiple signaling pathways regulate expression of MMPs, including the MAPK and NF-κB signaling pathways [15,16]. Thus, targeting these signaling pathways could prevent tumor metastasis and consequently reduce mortality.

In view of the therapeutic potential of OSCs in malignant diseases, abundant studies have focused on the effects of DATS on inhibiting tumor proliferation, inducing cell cycle arrest and apoptosis [17–20]. However, few of them investigate DATS in terms of tumor migration and invasion. In order to understand the mechanism of the anti-metastasis effect in TNBC, we investigated the inhibitory effect of DATS on cell migration, invasion and expression of MMP2/9 in TNBC cells and explored their underlying molecular mechanisms.

## Materials and Methods

### Materials and agents

DAS, DADS, and DATS (LKT Laboratories) were determined to be 98% purity by HPLC. Each compound was dissolved in 100% DMSO. 3-(4,5-Dimethylthiazol-2-yl)-2,5-diphenyltetrazolium bromide (MTT) was obtained from Sigma (St. Louis, Missouri). 8 μm Corning Transwell filter discs (for migration assay) were from Fisher Scientific (Nepean, Ontario, Canada). Actin-Tracker Green(for Immunofluorescence Staining) were from (Beyotime Biotechnology, Shanghai, China)

### Cell lines and cell culture

TNBC cell lines MDA-MB-231 and HS 578T were obtained from the Chinese Academy of Sciences Cell Bank of Type Culture Collection (CBTCCCAS, Shanghai, China). The breast cancer cell lines were cultured in DMEM supplemented with 10% fetal bovine serum, 100 μg/mL penicillin, and 100 μg/ml streptomycin and were maintained in an incubator with a humidified atmosphere of 95% air and 5% CO_2_ at 37°C.

### MTT assay

Cells were seeded in 96-well microplates (5,000 cells /well, 200 μL) and routinely cultured in a humidified incubator for 24h. The medium was aspirated off after a 24h pre-culture and exchanged for medium containing DATS at various concentrations ranging from 0 to 160 μM. Cells were then re-incubated for 6 and 24h. This assay was performed in triplicate. The medium was replaced with 100 μL of DMEM containing MTT solution (0.5 mg/mL). Cells were incubated for an additional 4 h. Then, 0.15 mL DMSO was added and the plates were shook for 10 min to dissolve the formazan crystals. Optical density of 96-well culture plates was then measured using an enzyme-linked immunosorbent assay reader at 490 nm. The optical density of formazan formed in untreated control cells was taken as 100% viability. The obtained optical densities from the treated wells were converted to a percentage of living cells (cell survival rate) against the control using the following formula: Absorbance of treated cells in the each well × 100/Mean absorbance of control cells.

### Cell Morphology and Immunofluorescence Staining

MDA-MB-231 and HS 578T cells were grown to 70%–80% confluence in 96-well culture plates. Then various doses of DATS and 0.5% DMSO were added to the media. Morphological changes were documented with a Carl Zeiss axio A1, at ×100 magnification at 24h time point.

MDA-MB-231 and HS 578T cells were grown to 70%–80% confluence in 6-well culture plates. Cells after 24h DATS treatment were fixed in 4% paraformaldehyde for 30 min, permeabilized in phosphate-buffered saline þ 0.1% Triton-X 100 for 10 min, stained by Actin-Tracker Green for 10 min. Then observed using a fluorescence microscopy at ×630.

### Wound healing mobility assay

5×10^5^ MDA-MB-231 and HS 578T cells were seeded into a 6-well plate and allowed to grow to confluent monolayer in complete medium. The monolayers were disrupted (i.e., wounded) by scraping them with a P200 micropipette tip, and cellular debris was dislodged by washing with PBS for 3 times.

Cell monolayers were incubated in the medium containing various concentrations of DATS for 24h at 37°C. At the indicated time (0, and 24h) after scraping, photographs of the exact wound areas were taken. Each dish was counted three times and the counts were averaged.

### Boyden chamber migration assay

Cell motility was tested in a Transwell Boyden Chamber (Corning Costar, Cambridge, Massachusetts) using a polycarbonate filter (8 μm pores). MDA-MB-231 and HS 578T cells (3×10^5^, 100μL) were re-suspended in medium containing various concentrations of DATS and carefully transferred into the upper chambers. The lower chamber was filled with 600 μL 20% FBS medium to attract cells in the upper chambers. The Transwell Boyden chamber was incubated at 37°C for 6 h. The cells on the upper side of the filter membrane were removed by wiping with cotton swabs after gentlely taking out of the filter from the chamber. The filter was fixed with 4% paraformaldehyde at 4°C and stained with 0.1% crystal violet stain solution. Cells penetrated the pore of the filter were fixed onto a glass slide. Cells in five randomly chosen microscopic fields (×200) of the lower slide were counted. Experiments were performed independently three times and the counts were averaged.

### Collagen invasion assay

In vitro invasion assays were performed under the same conditions as the Transwell chamber motility assays except the upper surface of the filter was coated with rat tail collagen. The rat tail collagen was stored at -20°C as a stock solution of 5 mg/ml. The working solution was generated by mixing rat tail collagen with 10 × DMEM and 1M NaOH in a 1.37:0.22:0.1 ratio. 70 μL was added in the upper chamber incubated at 37°C for 30 minutes. Then, DMEM was added on the surface of the collagen with continued incubation at 37°C for another 30 minutes and the medium was removed. MDA-MB-231 and HS 578T cells (3×10^5^, 100 μL) treated with various concentrations of DATS were carefully transferred on the collagen in the upper chambers. The lower chamber was filled with 900 μL 20% FBS medium to attract cells in the upper chambers. After the chamber was incubated at 37°C for 24 h, the filter was gentle removed and the cells on the upper side of the filter were wiped and fixed with 5% glutaraldehyde at 4°C and stained with 0.1% crystal violet stain solution. Finally, the cells were counted in five randomly selected microscopic fields (×200). Experiments were performed independently three times.

### Zebrafish metastasis experiment

Zebrafish used in this study were housed in the zebrafish facility at Model Animal Research Center (MARC), Nanjing University. The research protocol was approved by IACUC of MARC.

Fertilized zebrafish (Danio rerio) eggs were incubated at 28°C in Danieau’s solution and raised under standard laboratory conditions. Twenty-four hours post-fertilization, fish embryos were incubated with water containing 0.2 mM 1-phenyl-2-thio-urea (Sigma) to prevent pigmentation. Anesthetized embryos were transferred onto a modified agarose gel for microinjection. Before injection, tumor cells were labeled in vitro with 2 μg/mL of 1,1’-Dioctadecyl-3,3,3’,3’-tetramethylindocarbocyanine perchlorate (DiI, Fluka, Germany). Approximately 100–500 tumor cells were re-suspended in serum-free DMEM (Sigma) and 5 nL of tumor cell solution was injected into the perivitelline cavity of each embryo using an Eppendorf microinjector (FemtoJet 5247, Eppendorf and Manipulator MM33-Right, Märzhäuser Wetziar). Non-filamentous borosilicate glass capillary needles were used for the microinjection (1.0 mm in diameter, World Precision Instruments, Inc.). After injection, the fish embryos were immediately transferred into housekeeping water. Injected embryos were kept at 28°C and were examined every other day for monitoring tumor growth and invasion using a fluorescent microscope.

### Western blot assay

Whole-cell lysates were prepared with RIPA buffer containing protease and phosphatase inhibitors. Nuclear and cytoplasmic cell extracts were prepared using the NE-PER Nuclear and Cytoplasmic Extraction kit (Thermo, Rockford, USA). Equal amounts of cell lysates (50 μg) were loaded on 8 or 10% SDS-PAGE and transferred onto PVDF membranes. After the membranes were blocked, they were incubated with monoclonal antibodies against MMP-2 (Calbiochem, USA), MMP-9 (Calbiochem, USA), β-actin (Sigma-Aldrich, USA), and Integrin β1,3 (Millipore, USA). Goat anti-mouse horseradish peroxidase (HRP) and goat anti-rabbit HRP were purchased from Santa Cruz Biotechnology. NF-κB p65, phosphor-p65, phosphor-IκBα, IκBα (1:1000, Cell Signaling Technology), c-jun, c-fos (1:1000, Cell Signaling Technology), phosphor-ERK, ERK, phosphor-JNK, JNK, phosphor-p38, p38(1:1000, Cell Signaling Technology), phosphor-FAK Tyr 397, phosphor-FAK Tyr 925, phosphor-FAK Tyr 576/577, FAK (1:1000, Cell Signaling Technology), phosphor-src Tyr 527, non-phosphor-527, src (1:1000, Cell Signaling Technology), RhoA, Rac1/2/3 (1:1000, Cell Signaling Technology), Trx-1 (1:10000, Abcam), TrxR (1:20000, Abcam), GPADH (1:5000, Bioworld Technology) and Lamin B1 (1:5000, Epitomics) followed by incubation with horseradish peroxidase-conjugated IgGs (1:10000, Bioworld Biotechnology). Target proteins were developed with an ECL detection agent (Millipore, Braunschweig, Germany) and visualized with the ChemiDoc XRS system (Bio-Rad, Hercules, CA, USA).

### Quantitative real-time PCR assay

To confirm the results of RT-PCR, mRNA expression was also analyzed using an iCycler iQ Multi-Color Real Time PCR Detection System (Bio-Rad, Hercules, CA) with SYBR Green (Molecular Probe, Eugene, OR). The primer sequences used for real-time PCR and expected product size were described above. In brief, 1 μL of cDNA was added in a 24 μL reaction mixture containing 0.5× SYBR Green, 1× PCR buffer, 0.6 mM MgCl_2_, 0.4 mM dNTP, 0.5 mM primer sets and 0.625 U Amplitaq gold DNA polymerase (Applied Biosystems, Foster City, CA). The cycling conditions for all genes were as follows: preincubation at 50°C for 2min, 95°C for 4min, followed by 55 cycles of denaturation at 95°C for 20 s, annealing at 65°C for 30 s and extension at 72°C for 30 s. At the completion of cycling, melting curve analysis was performed to establish the specificity of the PCR product. The expression level of cDNA of each candidate gene was internally normalized using GAPDH. The relative quantitative value was expressed by the 2^-ΔΔCT^ method, representing the amount of candidate gene expression with the same calibrators. Each experiment was performed in duplicate and repeated three times.

### InnoZyme Gelatinase (MMP-2/MMP-9) activity assay

Briefly, 90 μL of either Gelatinase (MMP-2/MMP-9) Standard or sample (diluted in Activation Buffer) were added to designated wells and 10 μL Substrate Working Solution was added to each well. The wells were incubated at 37°C for 6 h and the resulting fluorescence was measured using a fluorescence plate reader set at an excitation wavelength of 320 nm and an emission wavelength of 405 nm.

### Dual luciferase reporter gene assay

Cells in 96 well culture plates were transiently transfected with 0.1 μg/well p-NF-κB-TA-Luc reporter plasmids (Biotime Biotech, Haimen, China). Transfection efficiency was normalized with renilla luciferase reporter plasmids. After 18 h post-transfection, cells were treated with indicated agents. Relative promoter activity was measured by dual-luciferase reporter (DLR) assay system using the Glomax 96 Microplate Luminometer (Promega, Madison, WI, USA).

### Statistical analysis

The results were analyzed by two-tailed student t-test SD using SPSS 11.0 (Aspire Software International, Leesburg, VA) and P-values were calculated. The difference was considered significant between two samples if P < 0.05.

## Results

### DATS inhibited cell viability and changed cell morphology in dose-dependent manners

**Fig 2:**
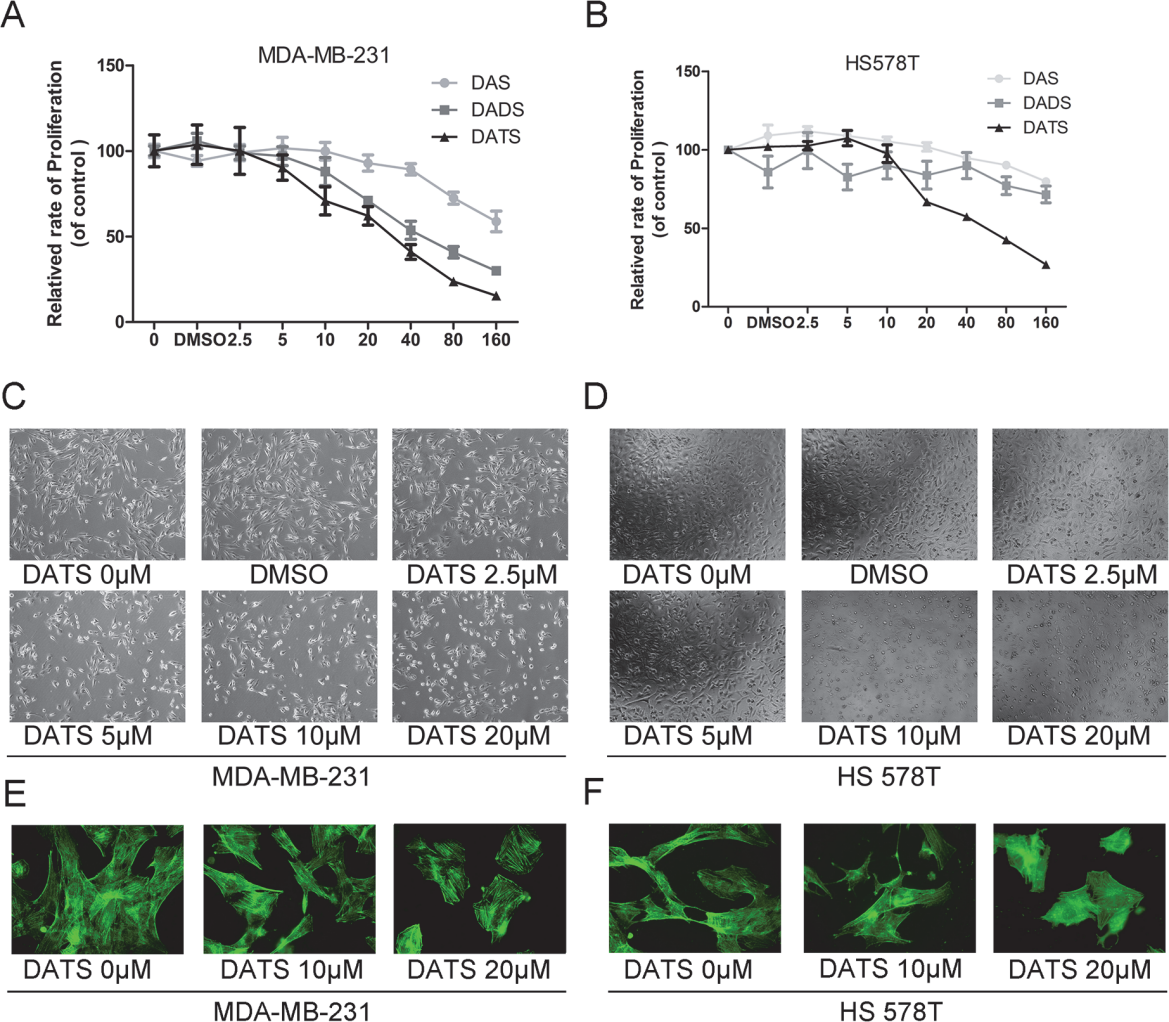
Garlic OSCs inhibited cell viability and changed cell morphology. An *in vitro* study was initiated by treating MDA-MB-231 (A) and HS 578T (B) cells with increasing doses of DAS/DADS/DATS (0, 2.5, 5, 10, 20, 40, 80, 160 μM)and DMSO(0.5%) for 24 h. The viability of the OSCs-treated cells was measured using the MTT assay. Results were expressed as a percentage of control, which was considered as 100%. Data were reported as mean ± SD and at least three separate experiments were performed. Cellular morphological changes in MDA-MB-231 (C) and HS 578T (D) cell lines were found in a dose-dependent manner after treated with DATS for 24 h when observed by a Carl Zeiss axio A1, at ×100 magnification. MDA-MB-231(E) and HS578T (F)no-treated or DATS-treated cells were immunostained with Actin-Tracker Greento observe F-actin stress fibers.

The number of sulfur atoms of OSCs has been speculated important to determine their chemical and biological activities [21]. The antiproliferative effects of DAS, DADS, and DATS were compared in human triple negative breast cancer MDA-MB-231 and HS 578T cells. In order to obtain the dose-response curve, we treated tumor cells with the increasing doses of DAS, DADS, and DATS (0, 2.5, 5, 10, 20, 40, 80, 160 μM) for 24h. The viability of the drug-treated cells was then measured using an MTT assay. The data showed that MDA-MB-231 and HS 578t (Fig 1A and 1B and S1 Table) cells were more sensitive to DATS than to DADS/DAS. We next investigated the cell morphology of MDA-MB-231 and HS 578T after treatment with different concentrations of DATS. Compared to controls, DATS-treated cells had smooth surfaces with obvious reduction of pseudopodia (Fig 2C and 2D). Moreover, in our experiments, Actin-Tracker Green staining showed that DATS at 10 and 20 μM could cause the spindle cells to round (Fig 2E and 2F).

### DATS inhibited cell migration in a dose-dependent manner

**Fig 3:**
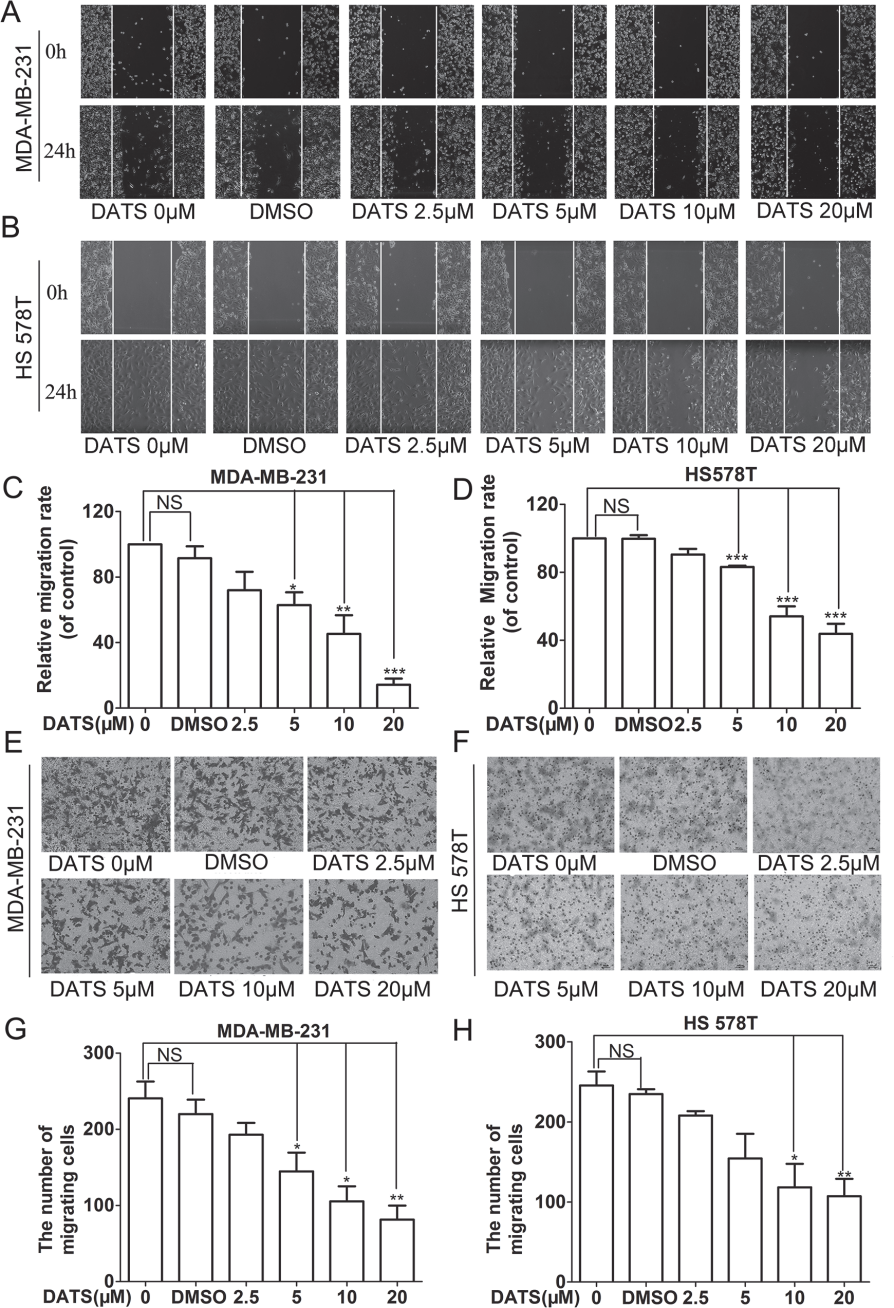
DATS inhibited migration in MDA-MB-231 and HS 578T cell lines. Confluent MDA-MB-231 (A) and HS 578T (B) cells were scratched and incubated at different concentrations of DATS (μM) and DMSO(0.1%). The area covered by migrating cells was recorded by phase-contrast microscopy connected to a digital camera at time 0 and 24 h. The wound closure area was calculated by measuring the diminution of the wound bed surface upon time using Image J software (C) and (D) Representative pictures of three independent experiments were shown. *, indicates P<0.05 versus no DATS group. MDA-MB-23 and HS 578T cells cultured in the upper well, DATS of indicated concentrations were put in the upper wells and add 10% FBS medium in lower wells, migration of the cells were determined by measuring the ability to pass through the filters. Migrated cells under the membrane after 12 hours on invert microscope (×200); The number of migration MDA-MB-231(E) and HS 578T(F)cells;each done in triplicate. *, P < 0.05and **, P < 0.01, compared with the control.

Cell mobility is a critical marker of metastatic potential in cancer cells. The motility of MDA-MB-231 and HS 578T human breast cancer cells was detected using a wound healing assay. Confluent monolayers of cells were scratched to form wounds and then cultured in the presence of various concentrations of DATS for 24h. Treatment of tumor cells with increasing concentration of DATS led to a dosage-dependent decrease in cell lateral transfer (Fig 3A–3D).

Additionally, the vertical migration of the MDA-MB-231 and HS 578T cells were examined. DATS showed a dose-dependent inhibitory effect on cell vertical migration (Fig 3E–3H) through the Transwell chamber (S2 Table).

### DATS inhibited invasion in a dose-dependent manner

**Fig 4:**
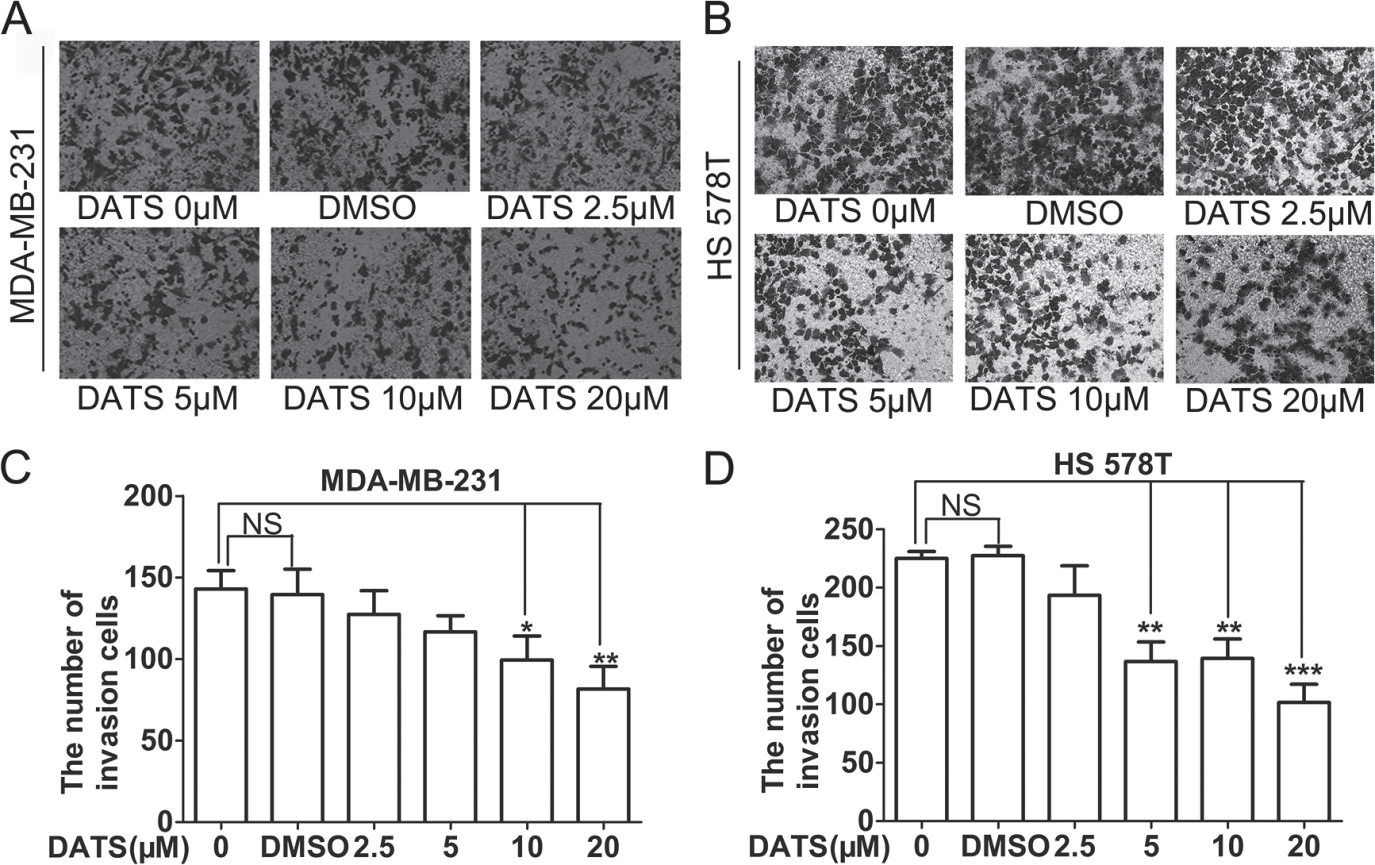
DATS inhibited invasion in a dose-dependent manner in MDA-MB-231 and HS578T cell lines. Approximately 1×10^6^ cells were seeded in the 24-well plate with cell culture inserts, the cells were treated with different concentrations of DATS (μM) and DMSO(0.1%) for 24 h to test invasion. Assays were performed as described in Materials and Methods. The results showed that DATS inhibited significantly cell invasion in a dose-dependent manner. *, indicates P<0.05 versus no DATS group. Data were shown as means ± SD from three independent experiments.

We next used Transwell matrigel invasion assays to determine whether DATS could decrease the cell invasive potential. Our results found that the invasive ability of MDA-MB-231 and HS 578T cells was decreased by DATS treatment in a dose-dependent manner (Fig 4 and S3 Table). Combining with the results of lateral transfer and vertical migration, these results show that DATS could inhibit the metastasis potential of triple negative breast cancer cells in vitro.

### DATS inhibited metastasis phenotype of MDA-MB-231 cell in zebrafish model

**Fig 5:**
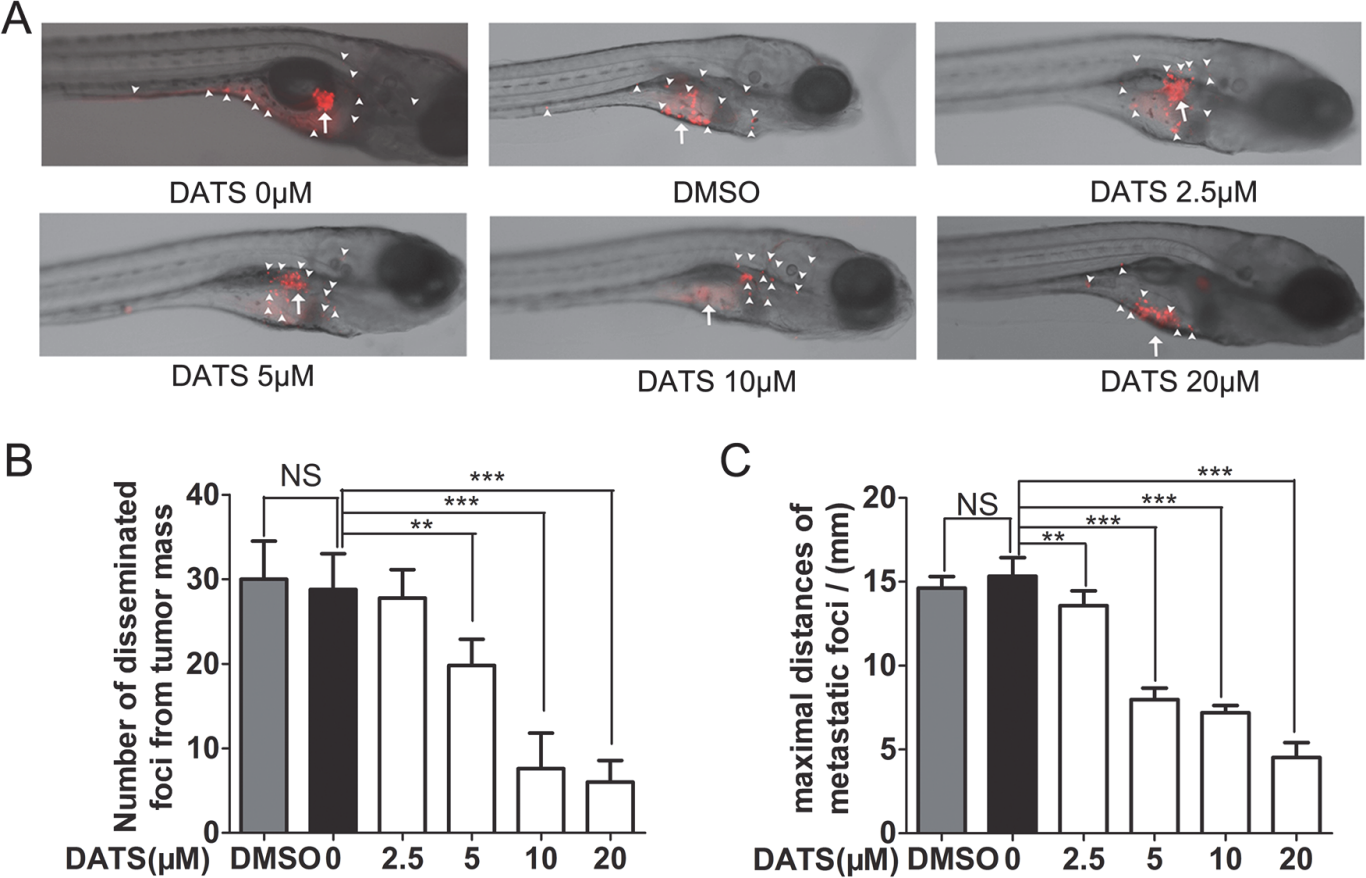
DATS inhibited the dissemination and metastasis of human tumor cells in zebrafish embryos. (A) DiI-labeled MDA-MB-231 cell were implanted in the perivitelline space and tumor cell dissemination were examined at day 6 post-injection. Arrows indicate primary tumors. White arrowheads indicate disseminated tumor foci. (B) Quantification of numbers of disseminated tumor foci (n = 10/group); (C) Averages of maximal distances of metastatic foci (n = 10/group).

A zebrafish tumor metastasis model was used to explore the anti-metastasis potential of DATS *in vivo*. The perivitelline cavity of 48 h post fertilization (hpf) embryos was injected with Dil stained MDA-MB-231 cells. We analyzed the model at 30 h post injection (hpi) by confocal microscopy. The spread of cancer cells could be observed throughout the zebrafish body. After exposure to different doses of DATS (0–20 μM) for 24h, the number of MDA-MB-231 cells disseminated foci and the maximal distances of metastatic foci were reduced by DATS in a dose-age manner (Fig 5 and S4 Table). This result was consistent with the results *in vitro*.

### DATS regulated the protein expression levels, mRNA levels, and enzyme activity of MMP2/9 involved in MDA-MB-231 cells metastasis

**Fig 6:**
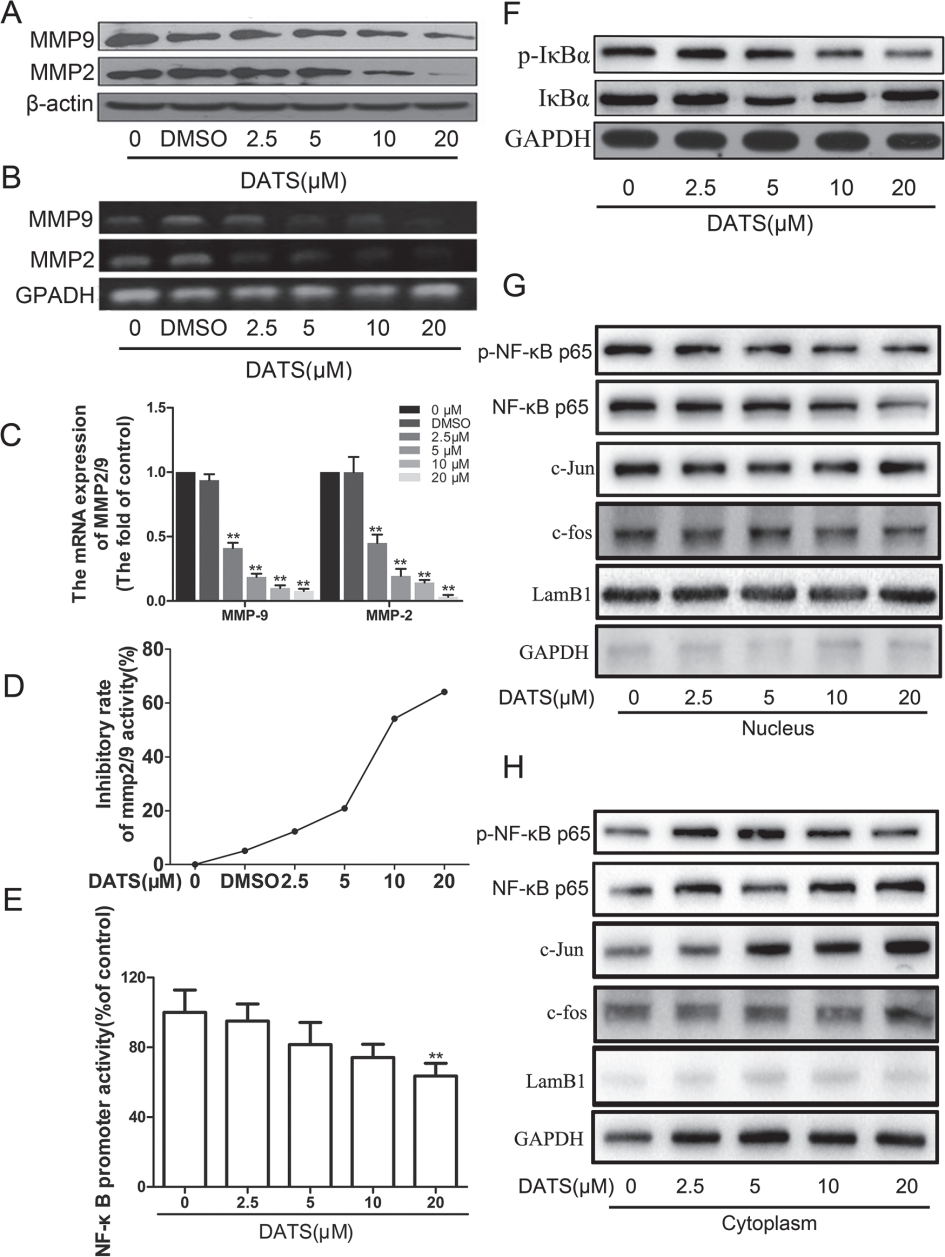
Critical role of NF-κB in DATS-induced transcriptional inhibition of MMP-2/9. MDA-MB-231 cells were treated with DATS (0, 2.5, 5, 10, 20 μM) DMSO (0.1%) and for 24 h and then subjected to (A) western blotting to analyze the protein levels of MMP-2/9; (B) Reverse Transcription-PCR and (C) quantitative real-time PCR to analyze the mRNA expression of MMP-2/9; (D) InnoZyme Gelatinase (MMP-2/MMP-9) Activity Assay to analyze the activity of MMP-2/9. (E) NF-κB promoter reporter assay to analyze the promoter activity of NF-κB. (F-H) Representative results of NF-KB protein levels and phosphorylation of IκBα by Western blot analysis.

MMPs play critical functions in cancer progression. MMP-2 and MMP-9 play an important role in tumor cell migration and invasion [22]. Therefore we investigated the effects of DATS on MMP2/9 expression. As shown in Fig 6A, DATS could dose-dependently down-regulate the protein expression of MMP-2 and MMP-9. Then the effect of DATS on the enzyme activity of MMP-2 and MMP-9 was ascertained by the Calbiochem InnoZyme Gelatinase Activity Assay kit (S1 Fig and S5 Table). As shown in Fig 6D, DATS treatment could lead to reduced gelatinase activity in a dose-dependent manner. MDA-MB-231 cells were treated with different dose of DATS for 24h and were then undergone to RT-PCR and real time-PCR to examine the mRNA levels. MMP-2 and MMP-9 levels were decreased considerably in a dose-dependent manner after treatment with various concentrations of DATS (Fig 6B and 6C and S2 Fig and S6 Table).

### NF-kB was essential for the transcriptional inhibition of MMP-2/9 by DATS

Promoter analysis of the MMP-2/9 has confirmed the cis-acting regulatory elements, including NF-κB[23]. To study the underlying mechanism of DATS mediated inhibition of MMP-2/9 at pre- transcriptional level, we performed dual luciferase reporter gene assay to investigate whether DATS could inhibit the transcriptional activity of transcription factors on MMP-2/9 (Fig 6E and S7 Table). To further investigate whether NF-κB was involved in the transcriptional regulation of DATS on MMP-2/9, we evaluated the effect of DATS on nuclear translocation of NF-κB. Treatment of MDA-MB-231 cells with 2.5 to 20 μM DATS attenuated phosphorylation of IκBα (Fig 6F). Furthermore, as shown in Fig 6G–6H, treatment of 2.5 to 20 μM DATS caused p65 to translocate from the nucleus to the cytoplasm. In addition, we found that DATS had a slight effect on AP-1 complex protein c-jun and c-fos. These findings indicated that DATS triggered transcriptional inhibition of MMP-2/9 by blocking NF-κB nuclear translocation.

### Effect of DATS on MAPK signaling pathway

**Fig 7:**
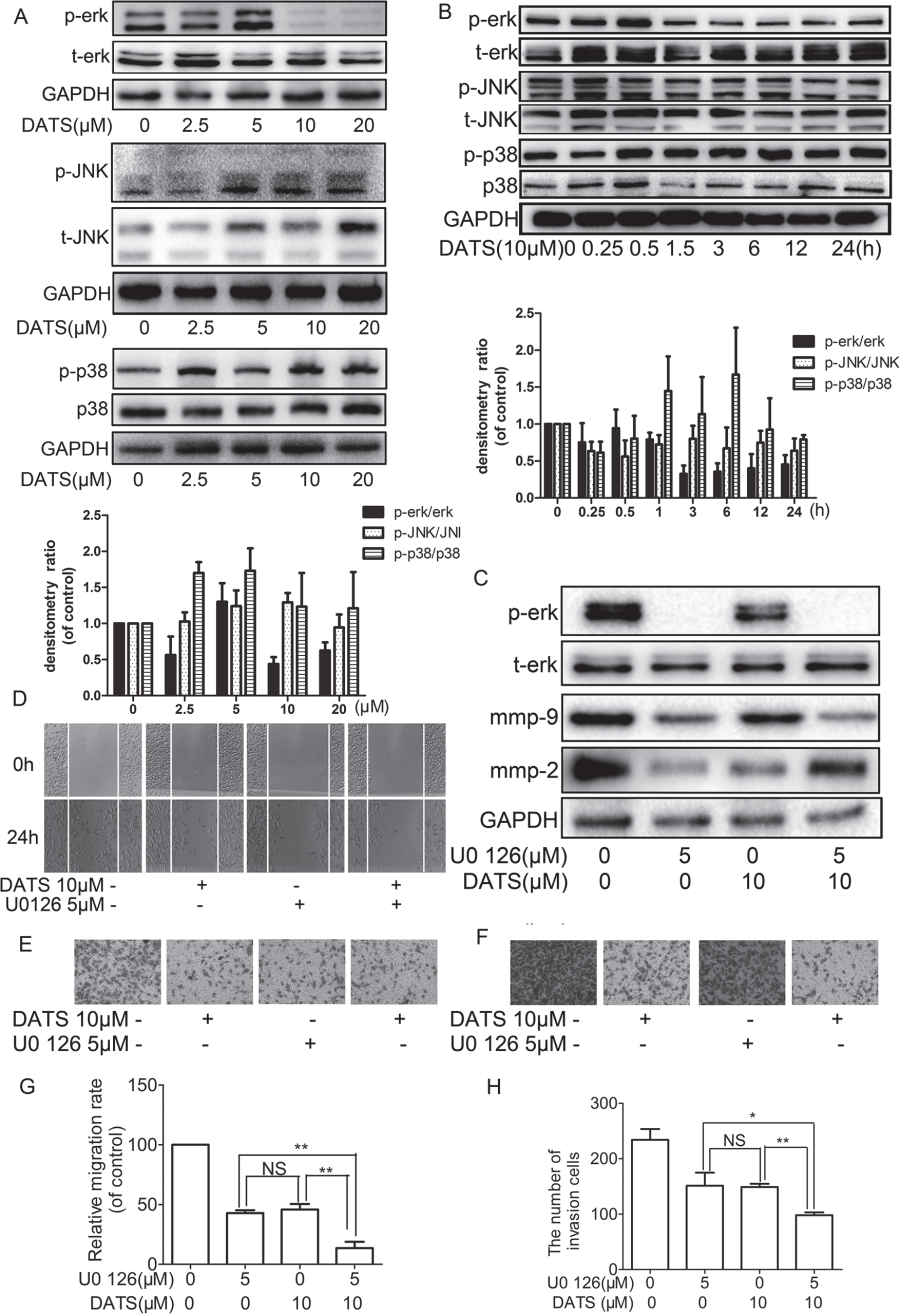
Effects of DATS on the MAPKs pathway. MDA-MB-231 cells were cultured in various concentrations of DATS (0, 2.5, 5, 10, 20 μM) for 24 h, and then the cell lysates were subjected to western blots with (A) anti-ERK1/2, anti-JNK, anti-p38 and (total and phosphorylated proteins) antibodies. (B) MDA-MB-231 cells were cultured in 10 μM DATS for different time periods (0–24 h), and then the cell lysates were subjected to western blots with anti-ERK1/2, anti-JNK, anti-p38 and (total and phosphorylated proteins) antibodies. (C) MDA-MB-231 cells were treated with 5 μM U0126 and incubated in the presence or absence of 10 μM DATS for 24 h, and then the cell lysates were subjected to western blots with anti-MMP-2/9, anti-ERK1/2 (total and phosphorylated proteins) antibodies. (D–G) MDA-MB-231 cells were treated with 5 μM U0126 for and incubated in the presence or absence of 10 μM for 24 h, MDA-MB-231 cells were then subjected to cell migration and invasion assay. The migration and invasion abilities of MDA-MB-231 cells were quantified by counting the number of cells that invaded to the underside of the porous polycarbonate. The values represented the means ± SD of at least three independent experiments.*P<0.05 as compared with the vehicle group.

Previous study had reported that the MAPK signaling pathway is involved in modulating the cancer metastasis and regulated MMPs in tumor cells. After evaluating the effect of DATS on the inhibition of cell migration and invasion, the effect of DATS on MAPK signaling pathway was investigated by western blotting to explore possible mechanisms. Western blotting showed that 10 μM DATS could reduce the expression of p-ERK in MDA-MB-231 cells in a dose- and time-dependent manner. However, phosphorylation of p38 and JNK 1/2 pathways were remain unaffected by DATS (Fig 7A and 7B and S8 Table).

### Effect of DATS on MMP-2/9 expression, migration and invasion of MDA-MB-231 cells with U0126

To further verify whether inhibition of MMP-2/9 expression, migration, and invasion by DATS on MDA-MB-231 through repression of the Erk1/2 signaling pathway, we used U0126, a known ERK1/2 inhibitor, to block ERK signaling pathway. Fig 6C showed that U0126 could strongly inhibit ERK1/2 phosphorylation and MMP-2/9 expression. When DATS was combined with U0126, the inhibition of DATS on MMP-2 was partly reversed, but the expression of MMP-9 was further decreased. Moreover, we found the stronger inhibition of MDA-MB-231 cell migration (Fig 7D and 7F) and invasion (Fig 7E and 7G), with sole treatment and with combined treatment (S9 Table). These results showed the inhibition of the EEK1/2 signaling pathways may be involved in reduced expression of MMP-2/9 and reduced malignant behavior in tumor cells.

## Discussion

Triple-negative breast cancer (TNBC) refers to breast cancers that do not express the genes for estrogen receptor (ER), progesterone receptor (PR) and the Her2/neu receptor and accounts for about 20% of breast cancers. Currently, chemotherapy is the only systemic therapeutic strategy available for TNBC patients. Management of TNBC is challenging because of a lack of targeted therapy, aggressive behavior and relatively poor prognosis. Since there are no specific treatment guidelines for TNBCs, they can only be managed by standard treatment. Anthracyclins, taxanes or a combination of broad-spectrum anticancer drugs are first line agents, but are prone to tumor resistance and cell toxicity [24–26]. TNBC is more metastatic and invasive than non-TNBC. It is essential to find novel anti-tumor agent. Garlic, as a medicinal and edible, has powerful anti-tumor properties. The organic sulfides (OCS) such as DAS/DADS/DATS have proven antitumor activity in different types of cancer cells including lung cancer, prostate cancer, breast cancer, and colorectal cancer [27–30]. In the present study, we found that the TNBC cell lines MDA-MB-231 and HS 578t were most sensitive to DATS. Previous research has shown that DATS induces apoptosis in the estrogen receptor positive cell line MCF-7, but the effect of DATS on metastasis of TNBC and the mechanisms were rarely reported. In our study, we found that DATS could change morphology of MDA-MB-231and HS 578T cells and therefore, may have anti-metastasis potential.

Eckhardt et al. have proposed that targeting tumor cell migration and invasion would be a good strategy to treat metastatic breast cancer [31]. In the present study, we determined that DATS inhibited MDA-MB-231 and HS 578T cell horizontal and vertical migration and inhibited invasion. In addition, we used a fluorescent zebrafish tumor metastasis model to prove that DATS had an effect on TNBC metastasis. The mechanism by which DATS inhibits invasion and migration is not yet clear.

The initiation of metastasis involves the interaction of tumor cells with the extracellular matrix (ECM), through the process of cell matrix adhesion and penetration out of the matrix [32,33]. The basement membrane is the largest barrier between a free malignant cell and the bloodstream, and it must be traversed before malignant cells can enter circulating blood or lymph. A large amount of studies have demonstrated that MMPs are critical for this process. MMPs are a family of zinc-dependent endopeptidases that play a crucial role in invasion and metastasis through the degradation of the ECM. In particular, MMP-2 and MMP-9 are collagenases that are crucial for tumor invasion and metastasis through degradation of collagen IV [34]. MMP-2 and MMP-9 are not new as therapeutic targets, but past clinical trials of early MMP inhibitors proved disappointing. The broad-spectrum MMP inhibitors previously trialed in breast cancer produced serious dose-limiting musculoskeletal toxicity, failed to reach therapeutic plasma levels, and did not extend survival—likely due in part to the inhibitors’ inability to distinguish among MMPs. In our present study, we found that DATS could inhibit MMP-2 and MMP-9 protein expression, as well as enzyme activity. Moreover, our results showed that the levels of MMP-2 and MMP-9 mRNA were decreased in MDA-MB-231 cells after DATS treatment.

The transcription of MMPs is regulated by upstream sequences, including motifs corresponding to NF-κB. It is a transcription factor shown to be significantly increased in TNBC tumors, which is consistent with the aggressiveness of these tumors [35]. Our present results demonstrated that DATS inhibited NF-κB transcriptional activity. In the cytoplasm, NF-κB is bound to a group of inhibitory proteins known as inhibitors of NF-κB (IκB). The accumulation of non-phosphorylated IκB prohibits the translocation of NF-κB from the cytoplasm to nucleus, resulting in inactivation of NF-κB and its downstream targets [36]. Our observations indicated that DATS inhibited NF-κB activity via inhibition of phosphorylation of IκBα and repressed nuclear translocation of p65. These seemingly contradictory findings indicate that the mechanism of MMP-2 and MMP-9 inhibition by DATS could be via modification of NF-κB. We predict that DATS may inhibit other cancer-related transcription factors and we are in the process of examining and identifying other signaling pathways and transcription factors altered by DATS (such as AP-1). Interestingly, DATS had no effect on c-fos and c-jun in our study, which normally regulate AP-1.

It is accepted that the activation of MAPK signaling pathway is important for regulating NF-κB activation and the subsequent activity of MMP-9 for cell lines in response to different stimulators [37]. Our results indicated that DATS had obvious effect on MAPK signaling especially in the inhibition of the ERK,. Moreover, the inhibition of MMP-2 by DATS was attenuated while MMP-9 was enhanced while blocking ERK signal. The various effects on the ERK might be caused by triggering multiple processes necessary for carcinogenesis [38]. However, the underlying mechanism needs further to be elucidated.

In conclusion, we showed that DATS suppresses the metastasis of the MDA-MB-231 and HS 578T breast cancer cell lines. We suggest that the anti-tumor properties of DATS may beneficial for the inhibition of metastasis by down-regulating the activities and expression of ERK/NF-κB /MMP-2/MMP-9. Garlic can be taken in large quantities in the human diet (2–10 g/day). Considering garlic has one to three percent of DATS and its derivatives,in our research, the concentration of DATS (i.e., 10 μM) should be achieved *in vivo* and be helpful for preventing or treating breast cancer.

## Supporting Information

S1 Fig The effect of DATS on enzyme activity of MMP2/9 of MDA-MB-231 cell. (TIF)

S2 Fig List of the primer sequences used in this study for Real Time PCR. (TIF)

S1 Table The effect of Garlic OSCs on breast cancer cell viability. (DOC)

S2 Table The effect of DATS on cell migration. (DOC)

S3 Table The effect of DATS on cell invasion. (DOC)

S4 Table The effect of DATS on metastasis phenotype of MDA-MB-231 cell in zebrafish model. (DOC)

S5 Table The effect of DATS on enzyme activity of MMP2/9 of MDA-MB-231 cell. (DOC)

S6 Table The effect of DATS on mRNA lever of MDA-MB-231 cell. (DOC)

S7 Table The effect of DATS on transcriptional activity of transcription factors of MDA-MB-231 cell. (DOC)

S8 Table Quantitative of the effect of DATS on MAPK protein lever. (DOC)

S9 Table The effect of DATS combined with U0126 on migration and invasion of MDA-MB-231 cell. (DOC)
