# Supplementary figures and images for: Antimetastatic Therapies of the Polysulfide Diallyl Trisulfide against Triple-Negative Breast Cancer (TNBC) via Suppressing MMP2/9 by Blocking NF-κB and ERK/MAPK Signaling Pathways
Source: PLoS One. 2015 Apr 30;10(4):e0123781. doi: 10.1371/journal.pone.0123781 (PMC4415928; doi:10.1371/journal.pone.0123781)

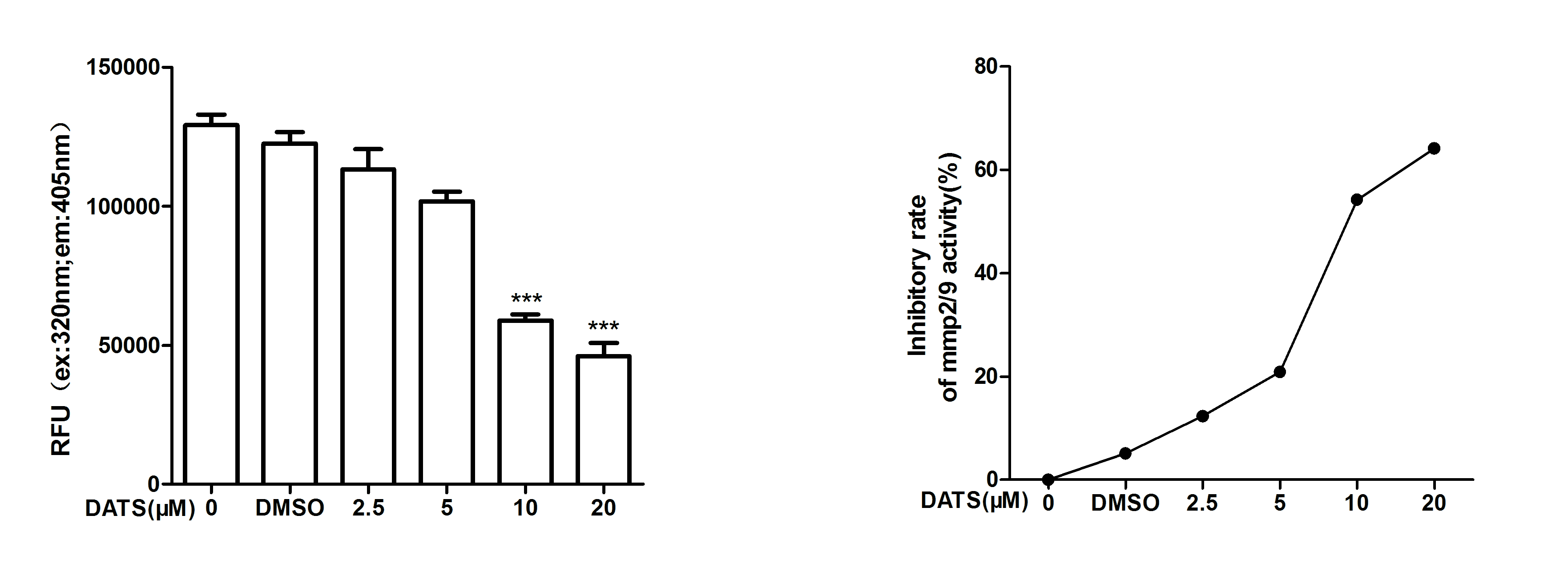

Supplement: S1 Fig — (TIF) [file pone.0123781.s001.tif]

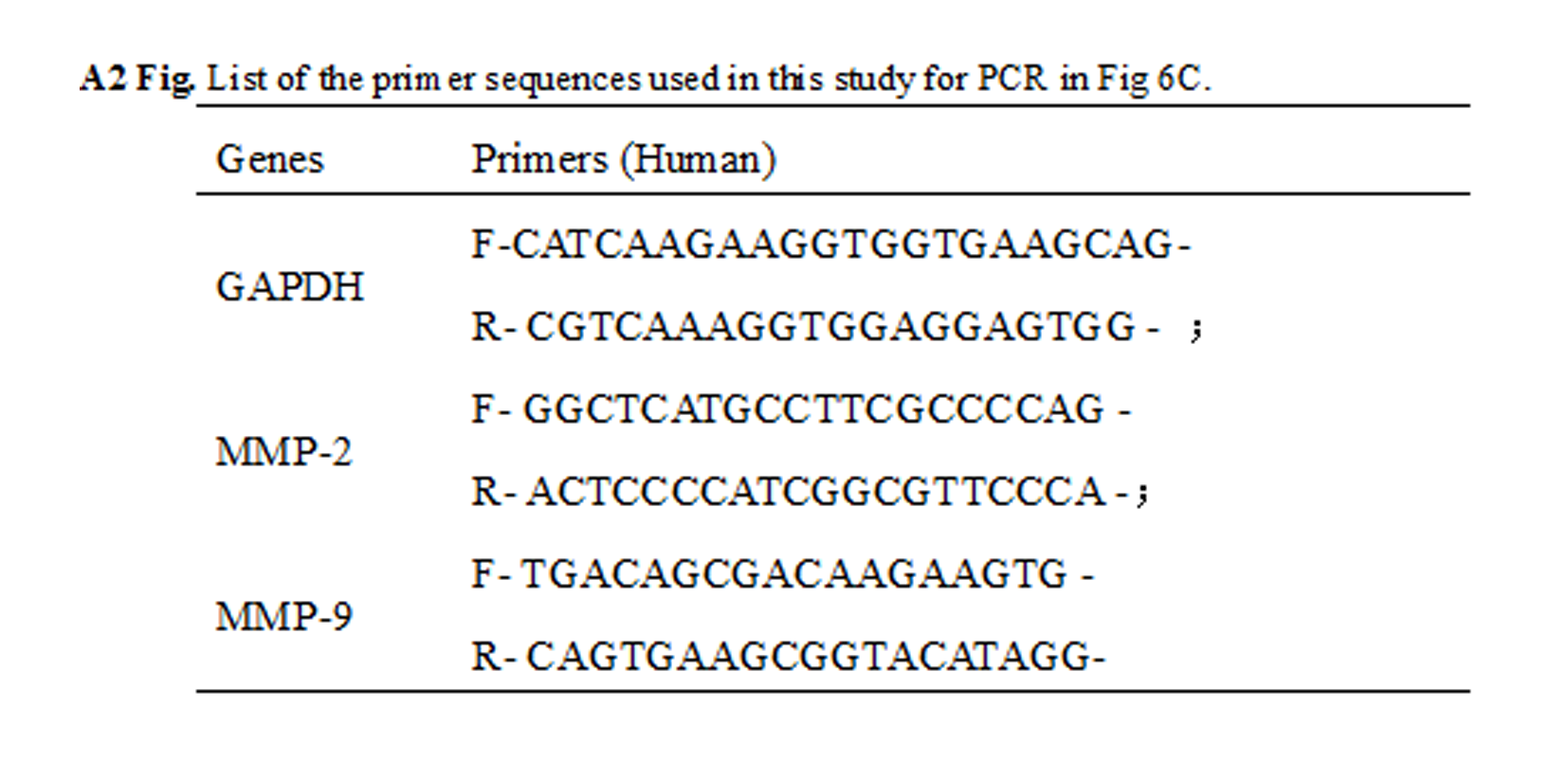

Supplement: S2 Fig — (TIF) [file pone.0123781.s002.tif]
