# Supplementary material for: Antimetastatic Therapies of the Polysulfide Diallyl Trisulfide against Triple-Negative Breast Cancer (TNBC) via Suppressing MMP2/9 by Blocking NF-κB and ERK/MAPK Signaling Pathways
Source: PLoS One. 2015 Apr 30;10(4):e0123781. doi: 10.1371/journal.pone.0123781 (PMC4415928; doi:10.1371/journal.pone.0123781)
Supplement: S1 Table — (DOC) [file pone.0123781.s003.doc]

**S1 Table.** The effect of Garlic OSCs on breast cancer cell viability shown in Fig 2(A-B),n=6

| Cell line | Relative growth rate | | | |
| --- | --- | --- | --- | --- |
|  | OSCs(μM) | DAS(%) | DADS(%) | DATS(%) |
| MDA-MB-231 | 0 | 100.00±3.88 | 100.00±2.85 | 100.00±9.27 |
|  | Dmso | 94.16±3.20 | 105.80±4.43 | 103.66±11.55 |
|  | 2.5 | 98.99±4.68 | 98.87±3.88 | 100.00±13.69 |
|  | 5 | 101.60±6.31 | 96.94±5.50 | 74.23±7.38 |
|  | 10 | 99.77±5.15 | 87.88±8.24 | 50.82±8.17 |
|  | 20 | 92.89±4.76 | 61.02±2.00 | 38.08±5.33 |
|  | 40 | 89.24±3.30 | 43.63±5.28 | 30.98±4.26 |
|  | 80 | 72.43±3.52 | 40.82±3.33 | 23.75±2.36 |
|  | 160 | 58.77±6.01 | 29.87±2.41 | 15.43±2.63 |
| HS 578T | 0 | 100.00±5.01 | 100.00±10.11 | 100.00±3.91 |
|  | Dmso | 111.38±10.18 | 85.83±24.83 | 101.99±5.04 |
|  | 2.5 | 135.66±20.81 | 99.48±28.23 | 102.59±6.63 |
|  | 5 | 133.59±19.49 | 82.57±20.06 | 107.43±12.03 |
|  | 10 | 124.02±13.65 | 90.08±21.23 | 97.58±13.92 |
|  | 20 | 127.41±25.82 | 83.86±21.58 | 66.75±4.14 |
|  | 40 | 92.89±19.03 | 89.85±20.47 | 57.36±3.10 |
|  | 80 | 66.60±12.67 | 77.14±13.89 | 42.54±3.12 |
|  | 160 | 76.47±22.19 | 71.53±13.15 | 26.95±4.05 |
