# Supplementary material for: Antimetastatic Therapies of the Polysulfide Diallyl Trisulfide against Triple-Negative Breast Cancer (TNBC) via Suppressing MMP2/9 by Blocking NF-κB and ERK/MAPK Signaling Pathways
Source: PLoS One. 2015 Apr 30;10(4):e0123781. doi: 10.1371/journal.pone.0123781 (PMC4415928; doi:10.1371/journal.pone.0123781)
Supplement: S2 Table — (DOC) [file pone.0123781.s004.doc]

**S2 Table.** The effect of DATS on cell migration shown in Fig 3,n=3

| DATS(μM) | Lateral transfer（%） | | Vertical migration（number） | |
| --- | --- | --- | --- | --- |
|  | MDA-MB-231 | HS 578T | MDA-MB-231 | HS 578T |
| 0 | 100±0.00 | 100±0.00 | 240.6±38.60 | 245.4±30.35 |
| D | 91.54±12.63 | 99.78±2.10 | 220.1±32.57 | 234.9±10.37 |
| 2.5 | 71.99±19.40 | 90.42±3.38 | 192.8±27.01 | 207.9±9.64 |
| 5 | 62.97±13.37^*^ | 83.12±0.80^***^ | 144.7±42.77^*^ | 154.4±53.07 |
| 10 | 45.26±19.96^**^ | 54.07±5.94^***^ | 105.2±34.42^*^ | 118.4±50.79^*^ |
| 20 | 14.20±6.58^***^ | 43.83±5.95^***^ | 81.4±32.15^**^ | 107.3±37.31^**^ |
