# Supplementary material for: Antimetastatic Therapies of the Polysulfide Diallyl Trisulfide against Triple-Negative Breast Cancer (TNBC) via Suppressing MMP2/9 by Blocking NF-κB and ERK/MAPK Signaling Pathways
Source: PLoS One. 2015 Apr 30;10(4):e0123781. doi: 10.1371/journal.pone.0123781 (PMC4415928; doi:10.1371/journal.pone.0123781)
Supplement: S3 Table — (DOC) [file pone.0123781.s005.doc]

**S3 Table.** The effect of DATS on cell invasion shown in Fig 4,n=5

| DATS(μM) | invasion（number） | |
| --- | --- | --- |
|  | MDA-MB-231 | HS 578T |
| 0 | 143.0±25.05 | 225.0±12.94 |
| D | 139.4±35.00 | 227.4±17.67 |
| 2.5 | 127.4±32.30 | 193.4±56.63 |
| 5 | 116.7±21.88 | 136.7±37.58^**^ |
| 10 | 99.3±32.94^*^ | 139.37±37.21^**^ |
| 20 | 81.6±30.86^**^ | 101.6±34.96^***^ |
