# Supplementary material for: Antimetastatic Therapies of the Polysulfide Diallyl Trisulfide against Triple-Negative Breast Cancer (TNBC) via Suppressing MMP2/9 by Blocking NF-κB and ERK/MAPK Signaling Pathways
Source: PLoS One. 2015 Apr 30;10(4):e0123781. doi: 10.1371/journal.pone.0123781 (PMC4415928; doi:10.1371/journal.pone.0123781)
Supplement: S4 Table — (DOC) [file pone.0123781.s006.doc]

**S4 Table.** The effect of DATS on metastasis phenotype of MDA-MB-231 cell in zebrafish model in Fig 5,n=10

| DATS(μM) | metastasis | |
| --- | --- | --- |
|  | Number of disseminated  foci from tumor mass | maximal distances of  metastatic foci / (mm) |
| 0 | 30.0±4.53 | 14.62±0.69 |
| D | 28.8±4.21 | 15.34±1.11 |
| 2.5 | 27.8±3.35 | 13.58±0.88^***^ |
| 5 | 19.8±3.11^**^ | 7.98±0.67^***^ |
| 10 | 7.6±4.22^***^ | 7.20±0.43^***^ |
| 20 | 6.0±2.55^***^ | 4.52±0.88^***^ |
