# Supplementary material for: Antimetastatic Therapies of the Polysulfide Diallyl Trisulfide against Triple-Negative Breast Cancer (TNBC) via Suppressing MMP2/9 by Blocking NF-κB and ERK/MAPK Signaling Pathways
Source: PLoS One. 2015 Apr 30;10(4):e0123781. doi: 10.1371/journal.pone.0123781 (PMC4415928; doi:10.1371/journal.pone.0123781)
Supplement: S5 Table — (DOC) [file pone.0123781.s007.doc]

**S5 Table.** The effect of DATS on enzyme activity of MMP2/9 of MDA-MB-231 cell in Fig 6D,n=3

| DATS(μM) | RFU（ex:320nm;em:405nm） |
| --- | --- |
| 0 | 129153.33±6439.53 |
| D | 122619.00±6944.04 |
| 2.5 | 113283.00±12398.62 |
| 5 | 101805.33±5943.37 |
| 10 | 58963.33±3565.97^***^ |
| 20 | 46057.67±8510.73^***^ |
