# Supplementary material for: Antimetastatic Therapies of the Polysulfide Diallyl Trisulfide against Triple-Negative Breast Cancer (TNBC) via Suppressing MMP2/9 by Blocking NF-κB and ERK/MAPK Signaling Pathways
Source: PLoS One. 2015 Apr 30;10(4):e0123781. doi: 10.1371/journal.pone.0123781 (PMC4415928; doi:10.1371/journal.pone.0123781)
Supplement: S6 Table — (DOC) [file pone.0123781.s008.doc]

**S6 Table. The effect of DATS on mRNA lever of MDA-MB-231 cell in Fig 6C.**

| The mRNA expression of MMP2/9  (The fold of control) | | |
| --- | --- | --- |
| DATS(μM) | MMP2 | MMP9 |
| 0 | 1.00±0.000 | 1.00±0.000 |
| D | 1.02±0.119 | 0.90±0.045 |
| 2.5 | 0.44±0.064^**^ | 0.41±0.042^**^ |
| 5 | 0.19±0.055^**^ | 0.28±0.026^**^ |
| 10 | 0.14±0.019^**^ | 0.10±0.020^**^ |
| 20 | 0.03±0.015^**^ | 0.07±0.016^**^ |
