# Supplementary material for: Antimetastatic Therapies of the Polysulfide Diallyl Trisulfide against Triple-Negative Breast Cancer (TNBC) via Suppressing MMP2/9 by Blocking NF-κB and ERK/MAPK Signaling Pathways
Source: PLoS One. 2015 Apr 30;10(4):e0123781. doi: 10.1371/journal.pone.0123781 (PMC4415928; doi:10.1371/journal.pone.0123781)
Supplement: S7 Table — (DOC) [file pone.0123781.s009.doc]

**S7 Table. The effect of DATS on transcriptional activity of transcription factors of MDA-MB-231 cell in Fig 6D.**

| DATS(μM) | NF-KB promoter activity(%of control) |
| --- | --- |
| 0 | 100.00±28.74 |
| 2.5 | 95.10±21.70 |
| 5 | 81.53±31.27 |
| 10 | 80.87±27.44 |
| 20 | 69.275088±25.23^**^ |
