# Supplementary material for: Antimetastatic Therapies of the Polysulfide Diallyl Trisulfide against Triple-Negative Breast Cancer (TNBC) via Suppressing MMP2/9 by Blocking NF-κB and ERK/MAPK Signaling Pathways
Source: PLoS One. 2015 Apr 30;10(4):e0123781. doi: 10.1371/journal.pone.0123781 (PMC4415928; doi:10.1371/journal.pone.0123781)
Supplement: S8 Table — (DOC) [file pone.0123781.s010.doc]

**S8 Table.** Quantitative of the effect of DATS on MAPK protein lever in Fig 7A-B.n=3

| DATS | % of control | p-erk/erk | p-JNK/JNK | p-p38/p38 |
| --- | --- | --- | --- | --- |
| 24h(μM) | 0 | 1.00±0.00 | 1.00±0.00 | 1.00±0.00 |
|  | 2.5 | 0.56±0.44 | 1.03±0.22 | 1.70±0.26 |
|  | 5 | 1.30±0.44 | 1.24±0.38 | 1.73±0.53 |
|  | 10 | 0.44±0.16 | 1.29±0.22 | 1.23±0.81 |
|  | 20 | 0.63±0.19 | 0.95±0.31 | 1.21±0.87 |
| 10μM(h) | 0 | 1.00±0.00 | 1.00±0.00 | 1.00±0.00 |
|  | 0.25 | 0.75±0.45 | 0.63±0.22 | 0.61±0.26 |
|  | 0.5 | 0.94±0.44 | 0.56±0.38 | 0.80±0.53 |
|  | 1 | 0.79±0.16 | 0.72±0.22 | 1.45±0.81 |
|  | 3 | 0.33±0.19 | 0.80±0.31 | 1.13±0.87 |
|  | 6 | 0.36±0.20 | 0.67±0.49 | 1.67±1.10 |
|  | 12 | 0.40±0.33 | 0.75±0.28 | 0.93±0.74 |
|  | 24 | 0.45±0.22 | 0.64±0.29 | 0.80±0.10 |
