# Supplementary material for: Antimetastatic Therapies of the Polysulfide Diallyl Trisulfide against Triple-Negative Breast Cancer (TNBC) via Suppressing MMP2/9 by Blocking NF-κB and ERK/MAPK Signaling Pathways
Source: PLoS One. 2015 Apr 30;10(4):e0123781. doi: 10.1371/journal.pone.0123781 (PMC4415928; doi:10.1371/journal.pone.0123781)
Supplement: S9 Table — (DOC) [file pone.0123781.s011.doc]

**S9 Table.** The effect of DATS combined with U0126 on migration and invasion of MDA-MB-231 cell in Fig 7E-H.n=3

| Group | blank | U0126 5μM | DATS 10μM | U0126 5μM+DATS 10μM |
| --- | --- | --- | --- | --- |
| Migration rate(%) | 100.00±0.00 | 42.85±4.16 | 45.90±7.81 | 13.57±9.10 |
| Invasion number | 234.1±33.71 | 151.5±40.63 | 149.0±10.00 | 98.1±8.83 |
